# Sensory trait variation in an echolocating bat suggests roles for both selection and plasticity

**DOI:** 10.1186/1471-2148-14-60

**Published:** 2014-03-27

**Authors:** Lizelle J Odendaal, David S Jacobs, Jacqueline M Bishop

**Affiliations:** 1 Department of Biological Sciences, University of Cape Town, 7701 Cape Town, South Africa

**Keywords:** Resting echolocation frequency, Neutral evolution, Mitochondrial DNA, Gene flow, Sensory ecology, Rhinolophidae

## Abstract

**Background:**

Across heterogeneous environments selection and gene flow interact to influence the rate and extent of adaptive trait evolution. This complex relationship is further influenced by the rarely considered role of phenotypic plasticity in the evolution of adaptive population variation. Plasticity can be adaptive if it promotes colonization and survival in novel environments and in doing so may increase the potential for future population differentiation via selection. Gene flow between selectively divergent environments may favour the evolution of phenotypic plasticity or conversely, plasticity itself may promote gene flow, leading to a pattern of trait differentiation in the presence of gene flow. Variation in sensory traits is particularly informative in testing the role of environment in trait and population differentiation. Here we test the hypothesis of ‘adaptive differentiation with minimal gene flow’ in resting echolocation frequencies (RF) of Cape horseshoe bats (*Rhinolophus capensis*) across a gradient of increasingly cluttered habitats.

**Results:**

Our analysis reveals a geographically structured pattern of increasing RF from open to highly cluttered habitats in *R. capensis*; however genetic drift appears to be a minor player in the processes influencing this pattern. Although Bayesian analysis of population structure uncovered a number of spatially defined mitochondrial groups and coalescent methods revealed regional-scale gene flow, phylogenetic analysis of mitochondrial sequences did not correlate with RF differentiation. Instead, habitat discontinuities between biomes, and not genetic and geographic distances, best explained echolocation variation in this species. We argue that both selection for increased detection distance in relatively less cluttered habitats and adaptive phenotypic plasticity may have influenced the evolution of matched echolocation frequencies and habitats across different populations.

**Conclusions:**

Our study reveals significant sensory trait differentiation in the presence of historical gene flow and suggests roles for both selection and plasticity in the evolution of echolocation variation in *R. capensis*. These results highlight the importance of population level analyses to i) illuminate the subtle interplay between selection, plasticity and gene flow in the evolution of adaptive traits and ii) demonstrate that evolutionary processes may act simultaneously and that their relative influence may vary across different environments.

## Background

In populations inhabiting heterogeneous environments adaptive trait divergence in the absence of gene flow is both predicted by theoretical models [1-3] and supported by a large number of studies demonstrating a generally negative relationship between levels of population connectivity and the degree of adaptive divergence in the traits under study [4-8]. This phenomenon is centred on the prediction that gene flow limits adaptive trait variation by homogenising gene pools that would otherwise diverge in response to selection under different ecological regimes [9-11]. The relationship is, however, clearly more subtle than this because empirical evidence also supports extensive adaptive variation in the presence of gene flow e.g. [12-15] highlighting the role of local selection gradients across different environments. If the selection gradient is steep enough to reduce immigrant fitness the homogenising effects of gene flow on trait variation are likely to be minimised [16]. Alternatively, if selection is weak and there are minimal costs to immigration, even nominal gene flow could constrain the evolution of trait variation and lineage divergence [17].

Undoubtedly there is a complex and dynamic relationship between selection, fitness and gene flow, but the interaction among these factors together with the role of trait plasticity is seldom considered in studies of adaptive population variation [18,19]. Phenotypic plasticity, environmentally mediated phenotypic changes without changes in allele frequency [20,21], has traditionally been viewed as a minor evolutionary process. This is so largely because environmentally induced phenotypic change does not influence the genes that an individual transfers to its offspring [19]; but see [22] for comment on the role of heritable epigenetic variation on phenotype evolution. Divergent selection cannot therefore act on genetic variants in a population and consequently phenotypic plasticity is thought to minimise rather than enhance selection [18]. Recently, however, there has been renewed interest in elucidating the role of plasticity in promoting diversification within and between populations and species [20,23-27]. This renaissance is due, in part, to the recognition that selection for adaptive plasticity can occur if it promotes successful dispersal and survival in different environments, increasing the potential for adaptive diversification under divergent selection regimes [19,28]. High gene flow between heterogeneous environments may then favour the evolution of increased phenotypic plasticity, over adaptive genetic divergence, because it would promote adaptation to new conditions within one or two generations [18,29]. Plasticity may in turn promote gene flow if dispersers are not selected against in their new environments [30]. Plasticity would allow a population or individuals to persist long enough in a new environment for selection to bring about the evolution of adaptation to the new environment by acting on the standing genetic variation among individuals [18,23].

Geographic variation in sensory traits i.e. traits used by organisms to perceive and respond to information about their environment [31] is well documented [32] and directly impacts on individual fitness e.g. via their role in resource acquisition and species and mate recognition [33]. Acoustic signalling for general communication as well as mate and food acquisition [34] is used by a range of organisms including fish [35], insects [36], anurans [37], birds [38] and mammals [39]. Extensive geographic variation in acoustic signals is common, and the role of both phenotypic plasticity [40,41] and sexually mediated selection have been implicated in promoting the evolution of population divergence [32,34]. In bats (Chiroptera) ultrasonic acoustic signalling in the form of echolocation is used for spatial orientation, prey detection and communication [42-44]. Bats echolocate over a wide range of frequencies [45] and the trait can be highly flexible both within [46,47] and between species [48]. This flexibility has been attributed to numerous factors including differences in foraging habitat [49,50], prey size [51], variation in body size [52], age [53] and sexual dimorphism [54]. In the horseshoe (Rhinolophidae) and leaf-nosed (Hipposideridae) bats a unique echolocation system has evolved that functions in both resource acquisition and intraspecific communication [55]. The signal design of horseshoe bats is characterised by long, high duty cycle calls which have a prominent constant frequency (CF) component preceded and followed by a brief frequency modulated (FM) component (45). During flight individuals compensate for Doppler shifts induced by their own flight speed by lowering the frequency of their emitted pulse. This ensures that the returning echo falls within the narrow frequency range of their acoustic fovea–a region of the cochlea with sharply tuned neurons sensitive to a unique frequency called the reference frequency [56]. Horseshoe bats are able to couple the frequency of the returning echo to their reference frequency independently of the size of Doppler shifts with extreme accuracy [57]. The reference frequency is always 150-200 Hz higher than the frequency these bats emit when stationary, often referred to as the ‘resting frequency’ (RF). Horseshoe bats typically forage for insect prey in narrow-space (i.e. highly cluttered) environments either on the wing (aerial hawking), or from a perch (flycatcher style) [58,59]. In both cases, the long CF component of their echolocation calls generate echoes from the flapping wings of flying insects that are characterised by amplitude and frequency modulations resulting in acoustic glints against a background of unmodulated echoes from background vegetation. Thus, the long CF calls emitted at a high duty cycle, together with Doppler shift compensation and the auditory fovea allow horseshoe bats to detect fluttering insects in cluttered space (reviewed in [60]). In horseshoe bats the RF is largely genetically determined [61]. However the peak frequency of juveniles is also partially influenced by that of their mothers [62,63] and the fine tuning of the frequency of the acoustic fovea of young horseshoe bats may have a learnt component via mother-to-offspring transmission [63,64]. Several recent studies investigating sensory variation in rhinolophid and hipposiderid bats [65-69] attribute observed variation predominantly to differences in diet [70] and environmental humidity (Humidity Hypothesis [71,72]). Such variation may be correlated with morphological features directly involved in echolocation production [47,73] and reception [74]. Sensory divergence may however also be an indirect result of adaptive changes in body size to prevailing environmental conditions (e.g. [71]). Among sympatric species call divergence may have evolved under regimes of social selection where character displacement maintains private bandwidths for intraspecific communication (Acoustic Communication Hypothesis [48,67,75]). Within a particular community, the echolocation frequencies used by any one species may therefore also be a consequence of the frequencies used by other species in that community [48,67]. Lastly, the physical structure of habitats (vegetation cover) can impose significant constraints on the range of frequencies bats use to successfully navigate within their environment and to detect prey, purely because of the physics of sound and sound transmission. Low frequency calls with longer wavelengths enable long-range detection of prey or other targets and are generally associated with open habitats. Conversely, higher frequency calls are more directional and provide greater resolution of targets, at least for bats using low duty cycle echolocation, at short detection ranges typical of highly cluttered habitats [45]. The Foraging Habitat Hypothesis [76] thus proposes that habitat clutter influences echolocation frequency and predicts that a gradient of increasing vegetation clutter selects for a gradient of increasing echolocation frequency. Most studies generally assume that observed variation in RF results from micro-evolutionary processes selecting for calls that are sharply tuned to the narrowband range of the acoustic fovea (± 1 kHz RF) [56,77,78]. Recent evidence, however, suggests that horseshoe bats and other high duty cycle echolocating bats exhibit some degree of temporal flexibility in RFs and associated changes in cochlear tuning [63,79] in response to both conspecifics [46,80] and local ambient noise [81].

More recently neutral evolutionary processes have become a focus of research centred on understanding echolocation divergence in bats [66,82]. Using the theoretical framework of population genetics, phylogeography and phylogenetics, the effects of gene flow and genetic drift on divergence in peak frequencies between divergent lineages has revealed relationships between echolocation divergence and e.g. female philopatry [66] geographic distance [7,69], and palaeoclimatic lineage divergence [82,83]. Here we use multiple data sets to investigate variation in echolocation frequency across the geographic range of the South African endemic Cape horseshoe bat, *Rhinolophus capensis*. We assess echolocation variation within a neutral evolutionary framework to (i) quantify the extent to which population genetic structure and gene flow contribute to variation in echolocation frequencies, and (ii) using a model-based approach explore the roles of habitat structure (Foraging Habitat Hypothesis [76]) and humidity (Humidity Hypothesis) in echolocation divergence. Our analyses reveal significant sensory diversification among *R. capensis* populations in the presence of extensive historic gene flow, and we discuss how selection, plasticity and gene flow may interact to characterize patterns of trait variation in this system.

## Methods

### Regional sampling of echolocation frequency and genetic variation

**Figure 1:**
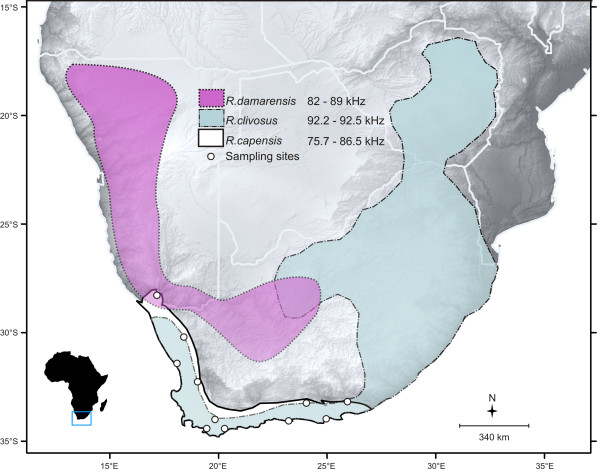
**Sampling localities and geographic distribution of sympatric horseshoe bats.** Geographic ranges *of R. capensis*, *R. damarensis* and *R. clivosus*. The ranges of RF of *R. damarensis*[87] and *R. clivosus*[77] in areas of range overlap with *R. capensis* are also indicated.

We measured echolocation parameters and collected tissue biopsies from populations across the distribution (Figure 1) of *Rhinolophus capensis*. Eleven populations were sampled across several major biomes in the region (Additional file 1: Figure S1, adapted from [84], no permission to reproduce maps was required) including Desert at the extreme northern edge, through Succulent Karoo and Fynbos in the centre of the distribution, and finally to Forest and areas of transition between Fynbos and neighbouring biomes (Albany Thicket, Nama-Karoo and Grassland) at the eastern limit of its range (Additional file 1: Figure S1). The geographic range of *R. capensis* is characterised by two distinct environmental gradients that influence regional rainfall patterns. The first is a latitudinal gradient of increasing aridity northwards. The second is a longitudinal seasonality shift from a predominantly winter to an aseasonal rainfall regime from west to east, and another shift to a summer rainfall regime at the extreme eastern edge of the distribution of the species [85] (Additional file 1: Figure S1). The resulting rainfall gradients translate into a clear habitat gradient from relatively open and sparse habitats in the north and west (Desert and Karoo Biomes), to more cluttered habitats in the east (Fynbos, Albany Thicket and Forest). Bats were captured at their roosts during the day with hand nets, or as they emerged from roosts at dusk using mist nets and/or harp traps. The age (adult or juvenile) and sex of each bat was recorded; juveniles were distinguished from adults by the presence of cartilaginous epiphyseal plates in their finger bones [86] and excluded from subsequent analyses. Individual body mass was measured. Seasonal and diurnal variation in body mass was controlled for by excluding pregnant females, sampling only in the southern hemisphere spring and summer and measuring bats only after their gut was emptied by keeping them overnight in soft cotton bags.

Echolocation calls were recorded from hand held bats positioned 10 cm in front of an Avisoft Ultrasound Gate 416 (Avisoft Bioacoustics, Germany) microphone connected to a laptop running Avisoft SasLab Pro software (sampling rate 500 kHz). Resting peak frequency (RF, where the CF component is stable and inter-pulse variation is low [47]) was recorded from hand-held individuals because it eliminates differences in peak frequency that may be due to Doppler shift compensation during flight [45]. RF is a reliable indicator of the reference frequency because the difference between the reference and resting frequency is stable in horseshoe bats (within 150-200 Hz) [60] Furthermore, when hunting from a perch rhinolophids emit their resting frequencies [88]. Recordings were slowed down by ten times and analysed using BatSound Pro software (Pettersson Elektronik AB, Sweden) with a sampling rate of 500 kHz. We measured the peak frequency (kHz) (frequency of maximum intensity) of the dominant second harmonic of the CF component determined from the fast Fourier transformation power spectrum (FFT = 1024, frequency resolution 684 Hz) with a Hanning window. To identify the typical echolocation parameters for each bat we calculated means of call duration, RF, lowest frequency of the FM component, bandwidth and inter-pulse interval from 5-10 high quality calls (amplitude of the signal at least three times higher than that of the background noise as displayed on the oscillogram). We chose the RF from an original call sequence that was most similar to the calculated mean parameters for all subsequent analyses. Thus the ‘true’ RF instead of a constructed statistical value was used in our analyses [73].

We also collected a tissue sample for DNA extraction and sequencing from most individuals for which we had echolocation recordings. Biopsy punches (3mm) were taken from the wing or tail membrane following [89]. Membranes were illuminated to ensure that no blood vessels were ruptured during sampling and tissues were stored in molecular grade (99%) ethanol at room temperature in the field, and at 4°C until extraction. Our capture, handling and tissue collection methods were approved by the Science Faculty Animal Ethics Committee (approval number: 2008/V18/LO) of the University of Cape Town. Total DNA was extracted using a DNeasy Blood and Tissue Kit (Qiagen) and stored at 4°C. We amplified a 520 base pair (bp) fragment of the mitochondrial d-loop using the primers N777 (5′TACACTGGTCTTGTAAAACC 3′) and E (3′ CCTGAAGTAGGAACCAGATG 5′) from [90] and [91] respectively. PCR conditions consisted of an initial cycle of 94°C for 5 minutes, followed by 35 cycles of 94°C, 50-55°C and 72°C each for 30 seconds and a final step of 72°C for 7 minutes. PCR products were checked on 1% agrose and gel purified using a Wizard SV Gel and PCR Clean-up System (Promega). Samples were sequenced in both directions using BigDye 3.1 chemistry on an ABI 3730 XL DNA Analyzer (Applied Biosystems). Chromatograms were edited and aligned using BioEdit v7.1.3.0 [92]. For each population the number of unique haplotypes, haplotype diversity (*Hd*) and nucleotide diversity (π) [93] were calculated using DnaSP v5.10.01 [94]. All unique haplotype sequences obtained in this study were deposited in GenBank (accession numbers: KF 175232-175270).

### Influence of non-genetic factors on echolocation divergence

We conducted a Kolmogorov-Smirnov test for normality, Levene’s test for homogeneity of variances and regressed means against standard deviations for all grouping levels to ensure the data met the assumptions of subsequent analyses.

To quantify variation in RF within and between populations we calculated the mean ± standard deviation (SD) and the coefficient of variation (CV = SD/mean × 100) of each population.

Various intrinsic (e.g. body size and sex) and extrinsic (e.g. habitat characteristics) factors can influence the evolution of geographic variation in acoustic signals across a species range. To tease apart the effects of these factors on RF divergence we first conducted a general linear model (GLM) with RF as the dependent variable and population, sex and their interaction as categorical predictors. We then used SPSS (version 21, IBM, USA) to conduct linear mixed models (LMM’s) performed with type III sums of squares to determine the ecological factors which best predict RF in *R. capensis*. Mixed models are robust to unbalanced designs and thus data from single-sex populations could be included [95]. LMM’s estimate the effects of both fixed and random factors to models of data that are normally distributed [95]. Our GLM results revealed significant differences between populations and sexes, but no significant interaction between them (see Results). Population and sex were therefore included as random factors to control for spatial clustering of samples and intrinsic sexual differences in RF [95]. Fixed factors included biome category and body mass. Even though the presence or absence of congeneric species may also influence RF variation across different populations of horseshoe bats, we were unable to explicitly test the Acoustic Communication Hypothesis for several reasons. First, the range of *Rhinolophus damarensis* (mean RF of 85.1 kHz in the region of overlap [87]), only overlaps with that of *R. capensis* at LS (Figure 1) and therefore its presence cannot be replicated across populations. Second, the presence of *R. damarensis* and the Desert Biome category are collinear and thus cannot be included in the same model [96]. Lastly, with the exception of the LS site, *R. capensis* co-occurs with a larger congeneric species, *Rhinolophus clivosus*, over much of its range. Five geographical lineages of *R. clivosus* corresponding to previously described sub-species based on genetic, acoustic and morphological differences have been identified, and of these, *R. capensis* overlaps with two lineages which echolocate between 92.5 and 92.2 kHz [83] (Figure 1). At these frequencies the RF of *R. clivosus* is unlikely to influence the RF of *R. capensis* and *R. clivosus* was therefore excluded from our analyses.

We employed a model selection approach based on Akaike’s information criterion (AIC) to determine which model, out of a range of candidate models, best explained RF divergence in *R. capensis*[97,98]. The model with the lowest AIC value was considered the most parsimonious and the difference in AIC scores (Δ_*i*_) were calculated to determine the likelihood that a given model was the best model relative to other candidate models [98]. A Δ_*i*_ value of zero indicates the best fit model; values up to two indicate models with substantial empirical support; values between four and seven indicate less support and models with values > 10 have essentially no support [98]. We also used Akaike weights (*w*_*i*_) to calculate the probability that a given model is the best among a candidate set of models. Thus, the best model has the lowest Δ_*i*_ and highest *w*_*i*_[98]. Once the best fit model was identified, we estimated the effects of each factor level on RF variation in *R. capensis* using the restricted maximum likelihood method. Then, to determine the importance of each variable included in the best model, we calculated the summed Akaike weight (*w*_+_) for all models containing that particular variable. The variable with the largest *w*_+_ is likely the most important variable in the model [99].

Our model selection results led us to explore the influence of habitat structure (typically vegetation cover) and body size on RF variation in greater detail. The Normalized Difference Vegetation Index (NDVI) was used as a measure of vegetation cover. NDVI is a suitable measure because it provides an estimate of above ground primary productivity [100] and it has been shown to be associated with a wide range of vegetation properties including photosynthetic activity [101] vegetation cover [102,103] and vegetation biomass [104]. Thus NDVI is commonly used to link vegetation dynamics to various aspects of animal ecology (reviewed in [105,106]). NDVI is a measure of the density of chlorophyll contained in vegetation and it is calculated as (NIR–RED)/(NIR + RED), where NIR is the near-infrared light, and RED is the visible-red light, reflected by vegetation and captured by the satellite. The values of NDVI range from -1 to 1, where negative values correspond to an absence of vegetation. Green and/or dense vegetation has high RED absorption together with high NIR, leading to high, positive NDVI values. In contrast, sparse vegetation absorbs substantially more NIR, leading to lower NDVI values [101]. Bare soils, snow and cloud have NDVI values close to zero [102,107]. We used the Expedited Moderate Resolution Imaging Spectroradiometer (eMODIS) Vegetation Indices dataset from NASA, which provides the maximum value for NDVI images for composites over a 10-day period at a resolution of 250 m from the year 2000 to present. Average NDVI values from a 20 km radius around each sampling site were extracted and compiled in ArcGIS 9.3.1 (ESRI®). Because echolocation frequency scales negatively with body size in horseshoe bats [52], variation in body size could cause concomitant changes in RF. Thus, to test whether RF variation is associated with differences in NDVI while controlling for the potential influence of body size, we used SPSS to conduct a hierarchal multiple regression analysis (HMRA). The first step of a HMRA is to add the independent variable that you wish to control for (in our case, body size). The second step examines the relationship between an independent (NDVI) and dependent variable (RF) while controlling for the effect of the first independent variable. We limited this analysis to males (n = 153) and evaluated the correlations between RF, body mass and NDVI as well as the change in the correlation coefficients between the model including body size and the model including NDVI but controlling for body size.

We also investigated whether there was a negative relationship between relative humidity and mean RF as predicted by the Humidity Hypothesis [108]. Relative humidity (%) data for each sampling site were obtained from the literature or from the nearest weather stations and provided by the South African Weather Service.

Like most other rhinolophids, *R. capensis* forage in or near highly cluttered habitats [67,109]. While the long CF portion of their echolocation calls allow the detection of fluttering prey against structurally complex backgrounds like dense vegetation [88], differences in vegetation cover between sampling sites could still influence the sound transmission properties of the echolocation call frequency used in each habitat. To better understand the effects of vegetation cover on variation in echolocation frequencies we calculated the mean detection distances for prey and vertical background targets (e.g. leafy vegetation edge) for each population according to the method developed in Stilz and Schnitzler [110]. This method depends on the dynamic range and frequency of the sonar system, local atmospheric conditions and target type. The dynamic range was calculated as the difference between peak intensity (dB SPL) at 1m (79.1 dB SPL for *R. capensis* at De Hoop; Jacobs and Parsons, unpublished data) and the auditory threshold of the bat (assumed to be 0 dB SPL for horseshoe bats [70,111]). We assumed the different populations had similar dynamic ranges. The different prey size categories tested in [110] were derived from Houston et al. [51] and included small, medium and large categories–all within the size range of prey consumed by *R. capensis* (2 mm-19 mm [67]). Mean minimum temperature (°C) data for each population were obtained from the nearest weather stations and provided by the South African Weather Service.

### Spatial population structure and patterns of gene flow

Geographic variation in phenotypic traits may be a consequence of neutral evolutionary processes, particularly when dispersal distances result in a pattern of predominantly nearest-neighbour gene exchange [112,113]. To better understand the role of random genetic drift in the evolution of RF variation we used a number of statistical approaches to (i) determine the degree to which genetic variation is spatially structured in *R. capensis* and (ii) quantify levels of historic gene flow among populations. We first explored the evolutionary relationships among mtDNA haplotypes to determine whether observed relationships reflected either the geographic sampling of populations or specific biome discontinuities across the range of the species by constructing a Neighbour-net network in SplitsTree v4.12.6 [114] using uncorrected ‘*p*’ distances. Network analysis allows reticulations among branches and is highly suitable for analysing evolutionary relationships among populations [115].

The spatial clustering of samples and distribution of genetic variation were also investigated using BAPS v6.0 [116] and Analysis of Molecular Variance (AMOVA) in GenAlEx v6.5 [117]. The AMOVA quantified partitioning of genetic variation within and among populations and biomes. The significance of variance components and *Φ*_*ST*_ (a measure of population genetic differentiation analogous to the fixation index *F*_*ST*_[118,119]) were estimated with 1000 random permutations. To test for the presence of discrete evolutionary lineages we estimated the most probable number of genetic clusters (K) among populations using a spatially explicit Bayesian clustering mixture model in BAPS. We performed spatial clustering for groups with K ranging from 4-11. The analysis was repeated ten times for each maximum K and the log marginal likelihood value for each genetic partition was evaluated.

To obtain estimates of historic gene flow among populations we used a maximum likelihood method based on the coalescent implemented in MIGRATE-N v3.3.2 [120]; MIGRATE uses an equilibrium model that estimates migration rates averaged across the coalescent history and simultaneously estimates Θ, the effective population size scaled by mutation rate where Θ = *N*_*e*_*μ*, together with pairwise migration rates summarised as *M* = *m/μ*, where *m* is the effective immigration rate per generation between populations. Banding data from European horseshoe bats reveal generally small home ranges where maximum dispersal distances rarely exceed 100km over the course of an individual’s life time [121]. If similar, dispersal in *R. capensis* likely occurs over relatively short distances. Thus we estimated Θ and *M* using a custom designed migration matrix where migration was only allowed between neighbouring populations. Populations separated by large geographic distances were not directly connected except in cases where unique haplotypes were shared. Starting values for all parameter estimates were initially obtained using *F*_*ST*_[122] and the following search parameters were used: 10 short chains with 500 000 gene trees sampled and 5000 trees recorded; three long chains with 50 million sampled trees of which 50 000 were recorded. The first 10 000 trees were discarded as burn-in and a static heating scheme with six temperatures and a swapping interval of one was used. The results were averaged over five replicate runs.

### Comparing echolocation divergence and genetic distance

Genetic divergence among populations is dependent on both the degree of physical isolation and levels of connectivity between them [123]. To test whether genetic and RF differentiation is associated with geographic isolation (isolation by distance) we used Mantel [124] and partial Mantel tests to explore the relationships between genetic structure, RF divergence and geographic distance [125]. Matrix correlations were calculated in GenAlex v6.5 [117] and XLStat (v2013, Addinsoft) with 1000 random permutations. The three matrices analysed were pairwise geographic distances (km) calculated as straight line distances from geographic coordinates using the program Geographic Distance Matrix Generator v1.2.1 (Ersts, Internet); genetic distance using Slatkin’s linearized *Φ*_*ST*_[126] and RF differences (kHz) among populations. Log-transformed geographic distances were regressed against genetic distance and peak frequency difference. Our partial Mantel test determined whether RF difference was associated with genetic distance while controlling for the effect of geographic distance (isolation by adaption).

## Results

### Effects of morphology, sex and ecology on RF divergence

**Figure 2:**
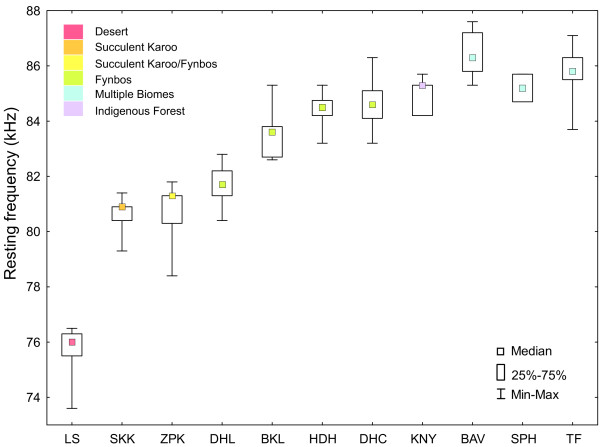
**Box and whisker plots of median resting frequencies across sampled populations of *Rhinolophus capensis*.** Colours represent biome category and a key to acronyms is given in Table 1.

We measured the RF of 248 individuals across multiple biomes (Additional file 1: Figure S1) and observed a clinal increase in mean RF across the distribution of *R. capensis* ranging from 75.7 kHz (LS) in the west, to 86.5 kHz (BAV) in the east (Figure 2, Table 1). Resting frequencies differed significantly among populations (GLM: *F*_18, 486_ = 120.5, *P* < 0.001; Tukey HSD tests: *P* < 0.005) with the exception of KNY which used similar frequencies to HDH and DHC (Tukey HSD tests: *P* > 0.05; Table 1). Sex significantly influenced echolocation variation within populations with females emitting higher frequencies than males (GLM: *F*_3, 172_ = 33.5, *P* < 0.001; Table 1). However, we detected no significant interaction between sex and population (GLM, *F*_18, 486_ = 1.1, *P* > 0.05), suggesting that the degree of sexual dimorphism in RF was identical across populations. The CV of call frequencies for each population was low and at most 1% (Table 1).

**Table 1:** Biome category and mean (± SD) body mass and RF for each sampled population

**Population**	**Biome category**	**GPS (decimal degrees)**	**Population mass (g) (*n*) (Mean ± SD)**	**Population RF (kHz) (*n*) (Mean ± SD)**	**CV (%)**	**Male RF (kHz) (*n*) (Mean ± SD)**	**Female RF (kHz) (*n*) (Mean ± SD)**
Lekkersing (LS)	Desert	-28.42, 16.88	(18) 13.2 ± 1.1	(18) 75.7 ± 0.8	1.06	(10) 75.5 ± 1	(9) 75.9 ± 0.5
Steenkampskraal (SKK)	Succulent Karoo	-30.98, 18.63	(22) 13.1 ± 0.9	(22) 80.6 ± 0.5	0.62	(11) 80.5 ± 0.6	(13) 80.8 ± 0.3
Zoutpansklipheuwel (ZPK)	Succulent Karoo/fynbos	-31.63, 18.21	(25) 12.3 ± 0.6	(25) 80.8 ± 0.8	1.08	Male only colony	
De Hel (DHL)	Fynbos	-33.08, 19.08	(10) 12 ± 0.45	(10) 81.5 ± 0.6	0.83	Male only colony	
Boskloof (BKL)	Fynbos	-34.39, 19.68	(15) 11.7 ± 0.5	(15) 83.4 ± 0.7	0.90	Male only colony	
Heidehof (HDH)	Fynbos	-34.62. 19.50	(12) 11.31 ± 0.5	(12) 84.5 ± 0.6	0.67	(6) 84.1 ± 0.5	(11) 84.8 ± 0.6
De Hoop (DHC)	Fynbos	-34.42, 20.36	(46) 10.4 ± 1.1	(46) 84.6 ± 0.7	0.90	(35) 84.1 ± 0.65	(23) 85.1 ± 0.6
Knysna (KNY)	Forest	-33.88, 23.00	(4) 11.6 ± 1.5	(4) 84.7 ± 0.7	0.7	(5) 84.8 ± 0.6	(2) 85.2 ± 0.7
Baviaanskloof (BAV)	Multiple biomes	-33.63, 24.24	(20) 10.8 ± 1.4	(20) 86.5 ± 0.7	0.90	(17) 86 ± 0.9	(10) 87 ± 0.5
Sleepy Hollow (SPH)	Multiple biomes	-33.96, 25.28	(2) 11.5 ± 0	(2) 85.2 ± 0.7	0.82	(1) 84.7	(1) 85.7
Table Farm (TF)	Multiple biomes	-33.283, 26.42	(36) 13.9 ± 0.8	(36) 85.8 ± 0.75	0.90	(19) 85.4 ± 0.65	(18) 86 ± 0.6

Mean (± SD) body mass and mean (± SD) resting frequencies (kHz) for populations (population acronyms in parentheses) and sexes are shown. Male only populations were ZPK, DHL and BKL. An equal number of males and females were used to calculate the mean population mass and RF. Multiple biomes occur at BAV, TF and SPH (combinations of Albany thicket, Nama Karoo, Fynbos and Savanna).

**Figure 3:**
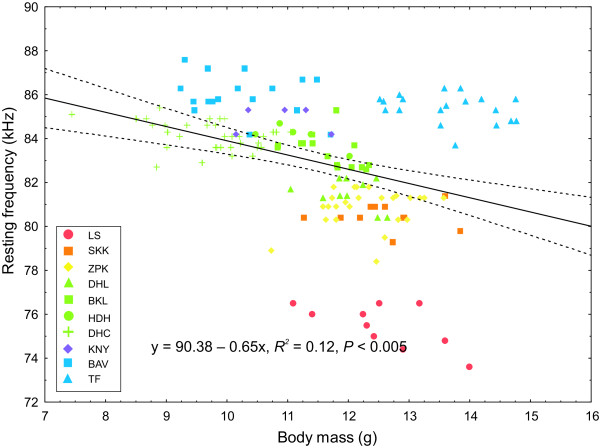
**The regression of mass (g) on resting frequency (kHz) for male *R. capensis *(n = 153) from 11 populations.** Colours represent the biome category of each population and shapes represent different populations. The key to the abbreviations of population names are given in Table 1.

**Figure 4:**
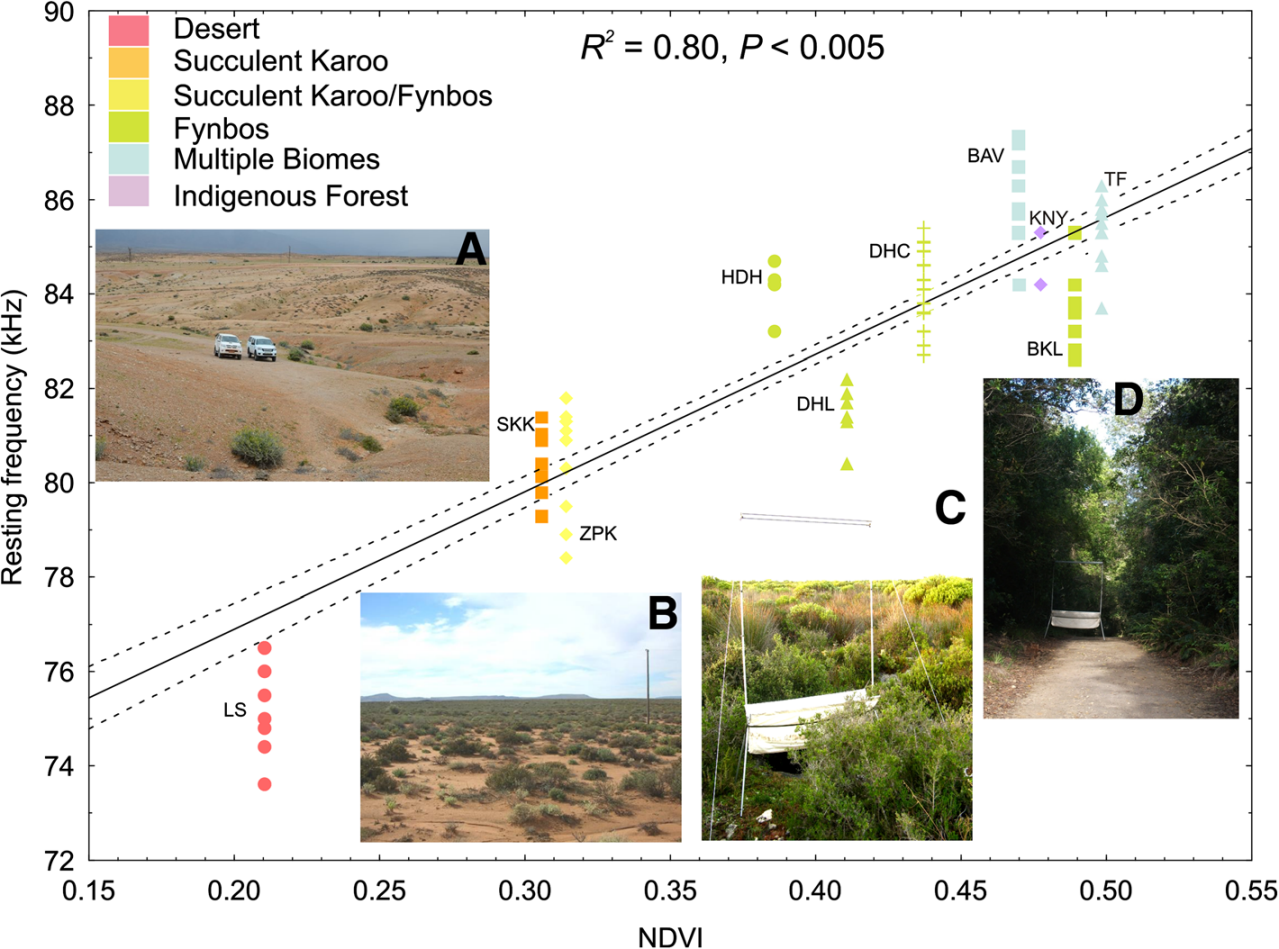
**The relationship between RF and NDVI (as a proxy for habitat clutter).** The regression of RF (kHz) and NDVI across populations of *R. capensis*. Habitat photographs show the vegetation cover and structure for each population, **A**: Lekkersing (Desert), **B**: Steenkampskraal (Succulent Karoo), **C**: De Hoop (Fynbos), **D**: Knysna (Forest). A key to acronyms used are given in Table 1.

The most parsimonious model explaining variation in RF included the factors biome category (*F*_5, 12_ = 40.3, *P* < 0.005) and body mass (*F*_1, 232_ = 10.4, *P* < 0.001) of which the former was the most important variable in the model (*w*_+_ biome = 0.99, *w*_+_ body mass = 0.95) (Table 2). Generally, bats from the Desert Biome use significantly lower frequencies than Succulent Karoo bats (point of reference selected by SPSS). Also, bats inhabiting the Forest Biome or regions comprised of multiple biomes use significantly higher frequencies (Table 3) because long–range detection is not an advantage in such habitats. In the first stage of our HMRA, body size significantly influenced RF (*R*^*2*^ = 0.121, *F*_1, 150_ = 20.6, *P* < 0.0001) but it only explained 12% of the variation in RF (Figure 3). Bats from LS use relatively lower RFs given their body size compared to other sampled populations. The inclusion of NDVI in the second stage of the regression model significantly increased the proportion of variance explained in the model (Δ *R*^*2*^ = 0.68, *F*_1, 149_ = 528.7.9, *P* < 0.0001), with NDVI accounting for 80% of the variation in RF (*R*^*2*^ = 0.80, *F*_2, 149_ = 310.9, *P* < 0.0001) after controlling for the effect of body size (Figure 4).

**Table 2:** Model selection results for three candidate models explaining variation in resting frequencies

**Model**	**AIC**	**Δ**_***i***_	**Weights (*w***_***i***_**)**
Biome	540.86	6.14	0.044
Body mass	588.46	53.74	2.04E-12
**Biome + body mass**	**534.72**	**0**	**0.95**

The most parsimonious model is highlighted in bold.

**Table 3:** Restricted maximum likelihood estimates and confidence intervals for the best-fit linear mixed effects model

**Parameter**	**Estimate (SE)**	***df***	**t-value**	**P- value**	**95% CI**
Intercept	80.85 (0.6)	11.8	125.9	<0.001	79.4, 82.3
*Biome*					
Desert	-4.9 (0.9)	11.7	-5.5	<0.001	-6.9, -2.9
Forest	4.1 (0.9)	13.3	4.4	<0.001	2.1, 6.1
Fynbos	2.8 (0.7)	12.1	3.8	<0.005	1.2, 4.4
Multiple biomes	5.4 (0.8)	11.5	6.9	<0.001	3.7, 7.1
Succulent/fynbos	.09 (1)	11.2	0.08	0.9	-2.3, 2.5
Succulent karoo^+^	0	0			
Body mass*	-0.17 (0.05)	231.9	-3.2	<0.001	-0.2, -.06

The best-fit linear mixed effects model describes resting frequency variation as a function of biome and body mass in *R. capensis*.

^+^Reference parameter.

*Centred variable.

We also found no relationship between relative humidity and RF (*R*^*2*^ = 0.04, *P* > 0.5) but we did discover significant differences in detection distances for large prey and vegetation edge (GLM: *F*_16, 306_ = 275, *P* < 0.01, Tukey HSD tests: *P*’s < 0.05; Additional file 2: Table S2), but not for small or medium sized prey (Tukey HSD tests: *P*’s > 0.05; Additional file 2: Table S2), between populations. There was, however, no difference in detection ranges associated with vegetation or large prey between ZPK, SKK and DHL and between TF and BKL (Tukey HSD tests: *P*’s > 0.05; Additional file 2: Table S2). Bats inhabiting more open habitats used lower frequencies and had longer detection distances than bats in more cluttered habitats, with LS bats (Desert Biome) having the longest detection distances of both large insects and background vegetation (Additional file 2: Table S2).

### Spatial population genetic structure and patterns of gene flow

A total of 39 unique mtDNA haplotypes were identified from 203 individuals (Additional file 3: Figure S2). Haplotype diversity ranged from 0.57 (SPH) to 0.88 (BKL) and the highest number of unique haplotypes (n = 12) was found in the Baviaanskloof area (BAV) (Additional file 4: Table S2). Most populations shared haplotypes with their nearest neighbours and a few haplotypes were shared between more distant populations (e.g. between ZPK and BAV) (Additional file 3: Figure S2). We identified three genetically isolated populations (i.e. LS, TF, and KNY) consisting largely of unique mitochondrial lineages (Additional file 3: Figure S2).

Complex network relationships characterise the evolutionary history of *R. capensis* populations sampled in our study. The neighbour-net network revealed numerous reticulations between haplotypes, suggesting several alternative evolutionary pathways among them ([127], Additional file 5: Figure S3). While the network recovered a number of clear clades these were not generally structured by biome or geographic proximity. Only haplotypes from the desert biome and populations occurring in regions where multiple biomes are connected formed discrete clusters (Additional file 5: Figure S3).

**Figure 5:**
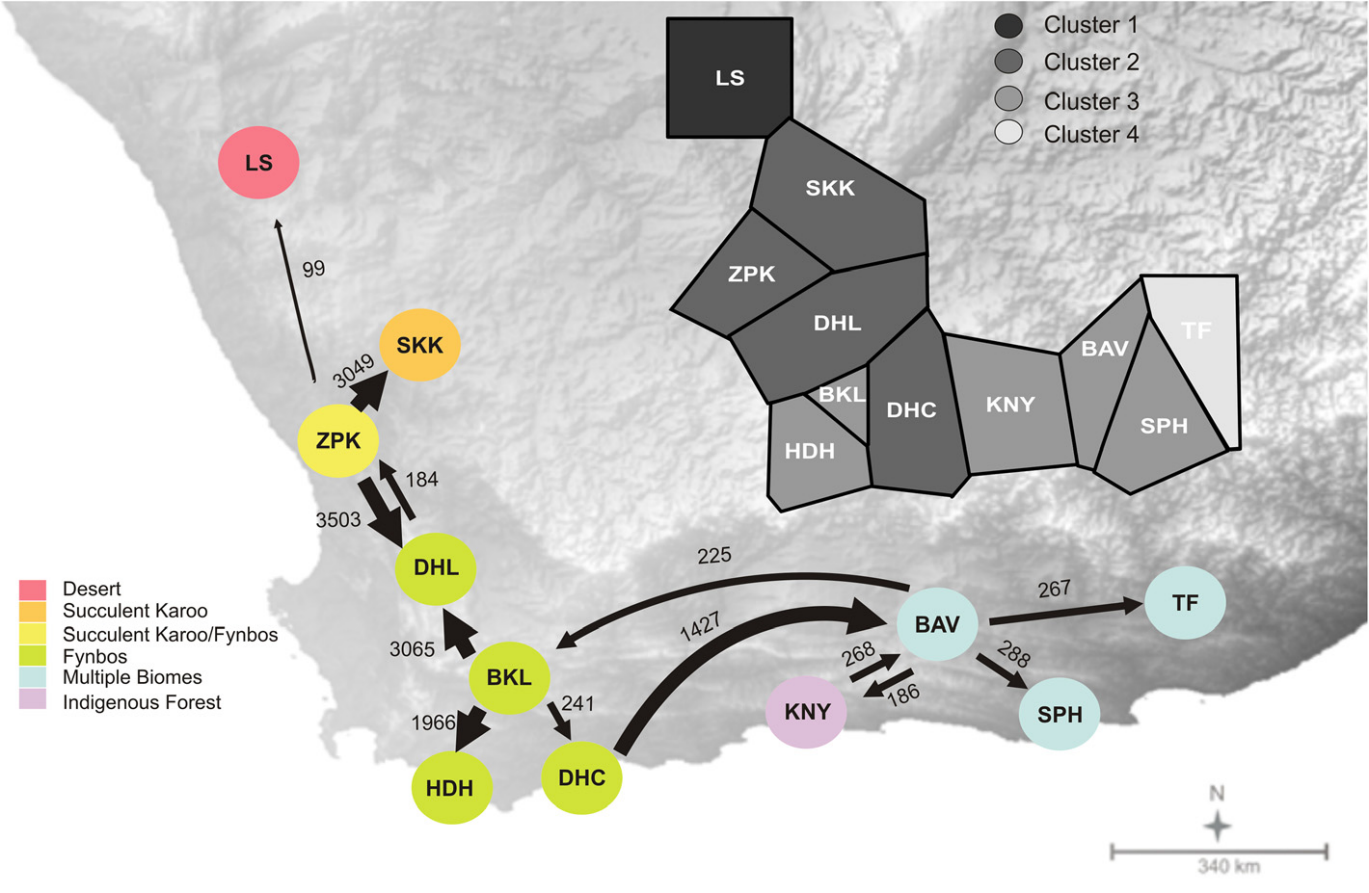
**Estimated migration patterns among populations of Cape horseshoe bats.** The thickness of the arrows indicates the relative migration rates; values indicate *M*, the number of immigrants per generation scaled by mutation rate. The 95% confidence intervals are provided in Additional file 6: Table S3. Inset: Voronoi tessellation showing the assignment of populations to four spatially defined genetic clusters. A key to acronyms used are given in Table 1.

Our investigation of hierarchal population genetic structure revealed significant partitioning of genetic variation at all three levels but most variation occurred within populations (*Φ*_*ST*_ = 0.54) rather than between populations (*Φ*_*ST*_ = 0.33) or biomes (*ΦST* = 0.13) (*P*’s < 0.005). Bayesian clustering identified four spatially explicit genetic clusters in our data (Figure 5). Only individuals situated at opposite ends of the species distribution (populations LS and TF), were assigned to unique clusters while the nine populations between them were assigned to two spatially structured clusters (Figure 5). Furthermore, estimates of historical migration rates (*M*) revealed generally asymmetrical patterns of gene flow between populations situated in different biomes (Figure 5, Additional file 6: Table S3). The main source populations were ZPK, BKL and BAV and gene flow generally occurred between neighbouring populations. There was also evidence for long distance gene flow from BAV to BKL (430 km straight line distance) and from DHC to BAV (370 km) but this occurred at relatively low levels (Figure 5). Together, these results support a pattern of relatively high regional connectivity and hence minimal fine-scale population structure in the *R. capensis*.

### Influence of genetic distance and geography

**Figure 6:**
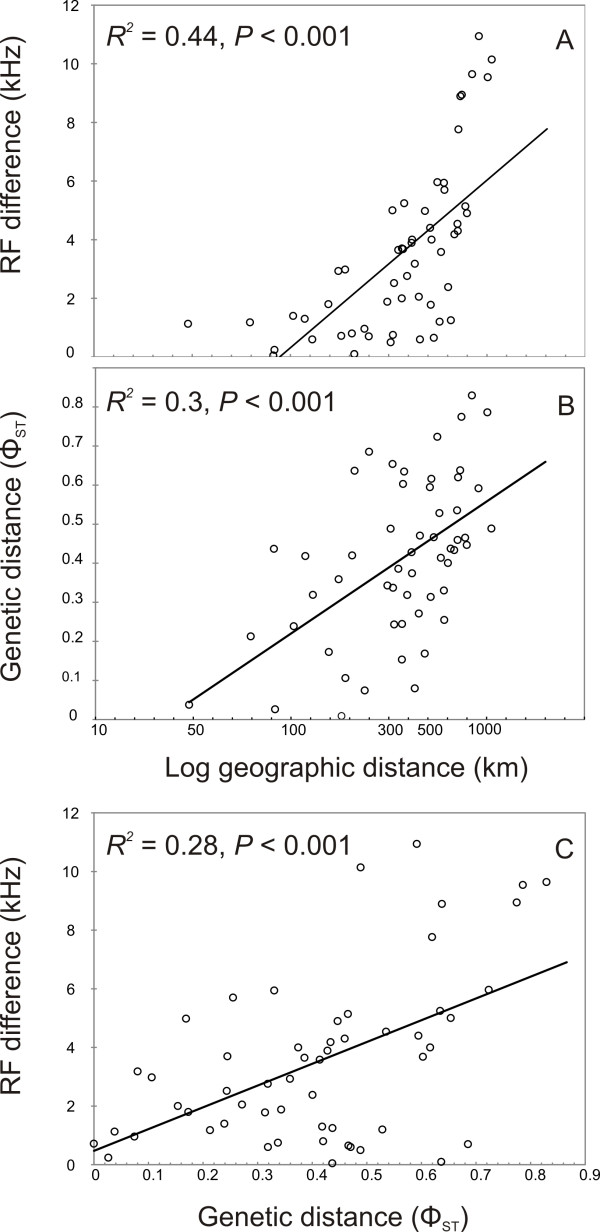
**Results from Mantel and Partial Mantel tests.** Pairwise geographic distance versus **A**: RF difference, and **B**: genetic distance; and **C**: genetic distance versus RF difference.

Pairwise differences in both RF and genetic distance were characterised by significantly positive relationships with geographic distance (Figure 6A, B). RF and genetic distance co-varied with geographic distance and followed a general model of isolation by distance (IBD). Geographic distance, however, explained only a relatively small proportion of the variation in RF and genetic distance (44% and 30%, respectively; Figure 6A, B). We also found a significant correlation between RF difference and genetic distance among populations (Figure 6C) and this remained significant when we controlled for the effect of geographic distance (Partial Mantel Test: *R*^*2*^ = 0.075, *P* < 0.001). Despite the significance of the correlation, genetic distance only explained a small proportion of the variation in echolocation frequencies (7.5%).

## Discussion

Our results reveal significant geographic variation in the resting echolocation frequencies (RF) of *R. capensis* despite substantial historical gene flow across the distribution of this species. Body size, genetic distance and geographic distance play minor roles in the evolution of geographic variation in RF in *R. capensis*. Further, despite a lack of strong geographic structure in mitochondrial lineages, biome was identified as the best predictor of RF. In support of this we found a significant relationship between RF and increasing vegetation clutter from west to east across the range of this species. Our results suggest that the evolution of geographic variation in RF in the face of homogenizing gene flow in *R. capensis* was probably influenced by selection for lower echolocation frequencies in less cluttered habitats. However, the relationship we found between RF and habitat type is solely correlative, and therefore a thorough comparison of foraging behaviours of bats between different habitats is required. Thus, alternative evolutionary processes e.g. selection for discrete frequency bands or phenotypic plasticity cannot be excluded at this stage.

### Disparities between RF and population genetic structure: trait diversification in the presence of gene flow

Studies investigating the various evolutionary forces shaping phenotypic and genetic divergence between populations often describe trait variation in the context of substantial population structure and limited gene flow [7]. While phylogeographic patterns of maternally (mtDNA) and bi-parentally (e.g. microsatellites) inherited genetic markers usually concur, discordance between phenotypic and genetic structure is also reported. This is usually attributed to demographic processes that characterize species, such as sex-biased dispersal [128] or secondary contact following historical isolation [47] (reviewed in [129]). Similar discordance has been reported in several European and Asian horseshoe bats either as a result of male-biased dispersal and female philopatry (e.g. *R. pumilus*: [66]) or historical introgression of mtDNA (*R. pearsoni*: [130]; *R. sinicus*: [131]; or nuclear genomes (*R. yunanensis* to *R. pearsoni*: [130]) between sister lineages. Despite recent advances in testing the evolutionary processes that shape contemporary population genetic structure in horseshoe bats (e.g. [132]), few studies have specifically evaluated sensory variation within a phylogeographic or population genetic framework in this genus [66,69]. In our study, we expected RF divergence in the *R. capensis* to reflect mtDNA structure given that (i) the fine tuning of the echolocation frequency of young horseshoe bats are partly learned from their mothers[63], (ii) female philopatry and male dispersal characterize other horseshoe bats studied to date [66,133], and (iii) the degree of RF divergence observed among populations (range: 1-11 kHz) is similar to that reported for other high duty cycle bats which have corresponding significant genetic structure among maternal lineages [77]. Our results instead reveal minimal genetic structure amongst *R. capensis* populations. A Bayesian clustering analysis identified four dominant genetic lineages which broadly reflect estimated patterns of regional gene flow that are not limited by biomes; notably only LS and TF (populations at the opposite extremes of the distribution of the species) are identified as unique genetic clusters (Figure 5). A number of mitochondrial haplotypes were also found to be shared between geographically distant populations; these may reflect long-distance dispersal events or the retention of common ancestral polymorphisms. Mitochondrial data clearly reveals a recent evolutionary history of complex reticulations in *R. capensis*, suggesting that gene flow and not incomplete lineage sorting is responsible for the observed genetic structure or lack thereof.

One caveat of our analytical approach is the statistical non-independence of our populations due to the substantial gene flow we detected between them [134]. Populations that are more closely related to one another or exchange higher numbers of migrants are likely to have similar phenotypic trait values irrespective of the local selection pressures they experience [134]. Ideally, the inclusion of our calculated migration matrices between populations in our ecological models would control for the effect of gene flow, but the development of effective computational methods to include the complex reticulate relationships among population’s remains challenging [134,135]. Nonetheless, the spatial distribution of the four dominant genetic lineages does not reflect the broad pattern of RF variation among populations of *R. capensis*. For example, we detected considerable gene flow between populations at SKK, ZPK and DHL, but no direct gene flow between these populations and the population at DHC, even though they were all assigned to the same genetic cluster (Figure 5). Bats from these genetically similar populations use RF’s ranging from 80.6-84.6 kHz. Thus the discordance between patterns of genetic and RF variation among *R. capensis* lineages suggest that gene flow does not significantly contribute to RF variation across the distribution of *R. capensis*, although this remains to be explicitly tested. Furthermore, although RF is also characterised by IBD, geographic distance only accounted for a minor proportion of the variation in RF (Figure 6A and B). Interestingly, RF divergence is also positively correlated to some degree with genetic distance, even after controlling for geographic distance (Figure 6C), suggesting that local environmental conditions may influence sensory differentiation in areas with some degree of restricted gene flow [136].

However, estimates of maternal gene flow support significant regional connectivity in the recent past and is at odds with the pattern of structuring in RF we observe across populations. This is in contrast to previous studies of high duty cycle bats where population genetic structure generally reflects the variation of sonar frequencies across often widespread species (e.g. *Pteronotus parnellii*: [77], *R. clivosus*: [73], *R. hildebrandtii*: [82], *R. rouxii*: [137]). As we discuss below, this anomaly may be a consequence of the complex interactions between gene flow, diversifying selection and environmentally mediated selection for phenotypic plasticity. Phenotypic plasticity can be advantageous if it results in the expression of different phenotypes that increase an individual’s fitness in diverse environments [19,138]. In this way plasticity can minimise the costs incurred from dispersal into environments with different selection regimes [18,29]. If plasticity influences RF diversification, it may lead to a situation in which selection does not significantly constrain gene flow among populations, leading to adaptive phenotypic variation in the presence of gene flow [18].

### Body size and habitat structure predict RF: influence of diversifying selection and adaptive phenotypic plasticity

The frequency of acoustic signals scales negatively with body size in a range of organisms [139-141] including bats [52]; larger bat species produce echolocation calls of lower frequencies than smaller bat species. This relationship also exists within *R. capensis* with larger bats generally using lower RF’s. However, body size only explained a minor proportion of the variation in RF because concomitant changes in body size and RF were not evident across all populations. For example, although BAV bats are not the smallest, they use the highest echolocation frequencies. In contrast, individuals in populations at both ends of the distribution of this species (LS in the west and TF in the east) were similar in size but echolocated at the lowest and second highest frequencies respectively (Table 1, Figure 3). This may explain why previous research on echolocation variation in the species did not support a relationship between body size and RF, and instead suggested the decoupling of echolocation divergence from the evolution of body size [73]. This result was likely an artefact of under sampling trait variation in *R. capensis* and highlights the difficulties of inferring evolutionary processes from data sets which inadequately sample the true distribution of a trait. Our results here suggest that the allometric relationship between body size and RF collapses in populations situated towards the edge of the range of this species. This is perhaps not unexpected given that range edges and ecotones provide novel environments to which species can become locally adapted, leading to significant phenotypic divergence of edge populations from those at the centre of the distribution of a species [142]. This may explain why populations at either end of the species’ range (LS in the west, and TF in the east) were identified as unique genetic clusters, while the nine sampled populations between them were assigned to only two genetic clusters (Figure 5).

Paleoenvironmental change from the Miocene to Pleistocene profoundly impacted the evolution of population divergence and speciation in a wide range of southern African taxa; several studies report a strong link between divergent genetic lineages and the biomes or ecogeographical regions of southern Africa for various organisms including reptiles [143], invertebrates [144] and small mammals [145,146] including bats [82,87]. In our study we did not find any clear population genetic divergence with topographical features. Neither did we find any support for the humidity hypothesis which proposes that divergence in RF is the result of selection against higher frequencies in humid environments [108]. Instead we found that bats in the Desert Biome used significantly lower frequencies than those occupying Forest and areas of transition between multiple biomes (Figure 4, Table 3). This clinal increase in RF across populations of *R. capensis* from west to east may be the result of the gradient of increasing vegetation cover and density from west to east (Figure 4). At LS in the west the vegetation was very sparse and consisted of low shrubs (< 1m in height). In the east the vegetation ranged from dense Fynbos to Forest (Figure 4). Thus, there is a steep habitat gradient between LS (genetic cluster 1) and its nearest neighbours (SKK and ZPK: genetic cluster 2), and selection may therefore dominate in this region. In contrast, plasticity may be favoured as an explanation for RF variation amongst the other populations because the habitat gradients are not as steep. A notable exception is that of the population at TF. At TF (genetic cluster 4) bats use similar RF’s to other populations situated in ecotones (genetic cluster 3), and yet appears to be relatively isolated genetically (Figure 5). TF is situated at the interface between aseasonal and summer rainfall zones [147] which, together with the intrinsic habitat heterogeneity of ecotones, may serve as a significant barrier to gene flow. This pattern has been observed for various other species in this region (e.g. four-striped mouse, *Rhabdomys pumilio*[145]; forest shrew, *Myosorex varius*[146]).

The positive correlation we found between RF and NDVI suggests that variation in the degree of habitat clutter might explain variation in RF. Across our sampling sites, the mean detection distance for large prey and background vegetation edge was significantly different between populations; bats occupying more open habitats have lower RF’s and thus longer detection distances than those in more cluttered habitats, allowing them to detect larger prey or background targets at greater distances. While this result was statistically significant, LS bats only had a 10 cm and 50 cm greater detection distance than their nearest neighbours for large prey and vegetation edge, respectively (Additional file 2: Table S1). Although all rhinolophids supposedly fly close to vegetation and may experience even relatively open habitats as cluttered, the 50 cm greater detection distances of the vegetation edge may be advantageous during orientation and commuting flight in the sparse vegetation of LS where the distance between clumps of vegetation are greater than in the Fynbos or the Forest (Figure 4). Further, prey density is also likely to be lower at LS and selection is likely to favour the evolution of lower RF’s that would allow the detection of larger prey at greater distances. There is sufficient empirical evidence to suggest that even rhinolophids, constrained by their echolocation to hunt in narrow space [58], display some degree of flexibility in the foraging habitat they exploit [148-151] or the foraging style they adopt (aerial hawking vs. perch hunting) [59,152] as a result of resource partitioning [150,153], habitat structure [148] or seasonal changes in prey resources [150]. At least one species of rhinolophid also appears to vary its echolocation frequency in response to different degrees of clutter [148]. Within the same nature reserve, greater horseshoe bats (*R. ferrumequinum*) use a variety of habitats with differing degrees of clutter and use significantly lower echolocation frequencies in relatively open habitats than in cluttered habitats [148]. It is likely therefore that *R. capensis* uses both aerial hawking and perch hunting styles to different degrees in the different habitats perhaps also altering its call frequency to deal with different degrees of clutter. The distinctly lower call frequency at LS could possibly be explained by the pronounced clutter gradient between LS and the other habitats occupied by *R. capensis*. However, our detection distance calculations must be interpreted with caution because different populations may have different echolocation call intensities, which could greatly influence the calculation of maximum detection distance [154].

Although differences in habitat structure provide a compelling explanation for different RF’s in *R. capensis*, an interesting anomaly needs to be accommodated; *R. damarensis* (forearm length 49.5 ± 1.7 mm; n = 20) is sympatric with *R. capensis* in the extremely arid area of LS but uses frequencies (mean ± SD = 85.4 ± 1.4 kHz; range = 82–89 kHz; [87]) as high as those used by *R. capensis* in highly cluttered habitats e.g. TF. It seems reasonable to assume that selection would have favoured similar call frequencies, and therefore detection distances, in *R. damarensis* and *R. capensis* where they are sympatric in the arid region of LS. However, it is possible that *R. damarensis* exploits a different foraging niche to *R. capensis* in this area of sympatry. Alternatively, this species may compensate by using higher intensity calls to achieve more or less the same detection distances as *R. capensis*[154]. Of course it is possible that the presence of *R. damarensis* at LS has selected for the low frequencies we observe in *R. capensis*. Observed frequencies are much lower in the LS population than in the other arid populations (Figure 4) despite similarities in body size to the other arid populations (Table 1). On the basis of its body size, *R. capensis* at LS should echolocate at approximately 82 kHz (Figure 3). Instead we observe a mean RF of 75.7 kHz. Bats at LS may have shifted their frequency below 82 kHz to avoid acoustic overlap with *R. damarensis* (82-89 kHz), and maintain effective intraspecific communication. However, the closest known roosts of these two species are 40 km apart and it is not known if the foraging areas of these two species overlap, i.e. if they are syntopic, as required by the Acoustic Communication Hypothesis.

Nevertheless, character displacement, mediated by some form of resource partitioning or local adaptation, in sonar parameters can contribute to the initial stages of lineage divergence by causing populations in sympatry with heterospecifics to diverge from their respective conspecific populations in allopatry. The result would be reduced gene flow between populations found in different selective regimes or heterospecific assemblages [155]. For example, Lemmon [37] showed that acoustic traits important for female preference and mate choice in populations of the chorus frog, *Pseudacris feriarum*, diverged to maximize differences from the heterospecific assemblage present, ultimately promoting reproductive isolation between conspecific populations via sexual selection. Ecologically adaptive traits can also promote divergence if divergence has a pleiotropic effect on reproductive isolation via assortative mating; so called ‘magic traits’ [34]. In *R. philippinensis* assortative mating has evolved between size morphs as a by-product of selection for different frequencies used to exploit different prey sizes [70]. The significant and consistent sexual dimorphism we observe in the RFs of *R. capensis* (female’s echolocate at higher frequencies than males: Table 1), may serve a role in sex-specific communication in the species. Recent experimental evidence reveals that horseshoe bats (*R. euryale* and *R. mehelyi*) and emballonurid bats (*Saccopteryx bilineata*) are indeed able to recognise the sex of conspecifics based on their echolocation calls [44,156]. Thus, the limited gene flow between LS and other populations may be a consequence of LS bats not effectively recognizing other *R. capensis* as potential mates. Divergence in RF may have under-appreciated consequences for the evolution of reproductive isolation via female preference for male RFs in different populations of horseshoe bats. Evaluating female preference in LS bats for local versus allopatric RFs may provide intriguing insights into the causes and consequences of sexual selection in horseshoe bats. Finally, because *R. damarensis* only occurs in sympatry with *R. capensis* at a single site (LS), we were not able to test whether habitat or the presence of *R. damarensis* offered a better explanation for RF differentiation in *R. capensis*. An experimental approach is required to test whether *R. capensis* has shifted its RF to avoid acoustic overlap with *R. damarensis*. Such an approach would evaluate whether *R. capensis* from LS responds differentially to the echolocation calls of heterospecifics and acoustically divergent conspecifics using playback experiments.

At a ‘local to regional’ scale geographic distance is not a significant barrier to gene flow in *R. capensis*, and the evolution of sensory divergence in the presence of this gene flow may also reflect a degree of adaptive phenotypic plasticity in RF. Despite the tight coupling between RF and the acoustic fovea in high duty cycle bats [45], empirical studies have shown that species are able to shift their RF’s in response to both neighbouring conspecifics (maximum shift 3.9 kHz: [46]) and different ambient noise conditions (maximum shift <0.5 kHz: [81]). Such small shifts in frequency may explain the range of RF variation in the southern and eastern populations of our study (approximately 3 kHz) where plasticity in response to slightly varying degrees of vegetation clutter towards the east might occur. However, it is unlikely to explain the 9 kHz shift in the Lekkersing bats. Nonetheless, it appears that southern and eastern populations of *R. capensis* use RF’s within the best hearing range of the acoustic fovea of their nearest neighbours, possibly facilitating gene flow and promoting relatively flexible RF’s in these populations. While small shifts in the acoustic fovea and its corresponding reference frequency are possible in high duty cycle bats [46,81], we do not know the precise limits of the flexibility of the acoustic fovea. Long-term experimental studies evaluating the change in RFs in response to the RFs of bats from acoustically divergent populations may shed light on the degree of plasticity in this system. Alternatively, the relative influence of plasticity versus selection can be evaluated indirectly. For example, selection may better explain our observations if variation in functional genes involved in hearing co-varies with RF variation across populations. Recent studies reveal a wide range of candidate hearing genes which show strong signals of ancestral positive selection in the evolution of echolocation in bats and cetaceans [157,158]. Selection may also be favoured as an explanation for variation in RF if there is a strong correlation, independent of variation in body size, between RF and morphological features directly involved in echolocation production and emission (such as dorsal nasal chambers) [47]. Previous research investigating morphological correlates of RF in *R. capensis* revealed that RF is best predicted by nasal chamber length [73], but a thorough evaluation of skull morphology variation across the distribution of the species is required. The social life of bats may also influence the relative roles of diversifying selection versus plasticity across the distribution of a species. If bats are able to recognise conspecific calls from a range of acoustically divergent populations this might suggest that selection for some degree of plasticity in the trait is also favoured. Classic playback experiments can be used to assess the sensitivity of individuals to the range of frequencies exhibited by a species. These two hypotheses are clearly not mutually exclusive; and their relative influences across the highly heterogeneous environments of *R. capensis* certainly merit further attention.

## Conclusions

This study reveals significant sensory trait variation in the Cape horseshoe bat despite substantial historical gene flow. While genetic and geographic distances do influence sensory variation to some extent, our results suggest that differences in habitat complexity across the range of the Cape horseshoe bat may be the dominant driver of sensory differentiation in this system. Classical divergent selection together with some degree of phenotypic plasticity may be responsible for RF variation in the presence of gene flow. However, an investigation of the variation in foraging behaviour within and between populations of the Cape horseshoe bat is required to support our results. Nonetheless, our findings highlight the importance of population level analyses to elucidate the complex interactions among selection, plasticity and gene flow in the evolution of adaptive trait variation and reveal a number of interesting avenues for future research into the evolution of this remarkable sensory system.

## Competing interests

All authors have declared that no competing interests exist.

## Authors’ contributions

LJO, DSJ and JMB conceived of and designed the study and carried out the fieldwork. LJO generated the molecular data, and conducted all genetic and ecological analyses with input from JMB and DSJ. All authors contributed to the writing of the manuscript and read and approved the final manuscript.

## Supplementary Material

Additional file 1: Figure S1 Map of the biomes of South Africa together with the geographic locations of the 11 populations of *Rhinolophus capensis* sampled in this study Biomes are from Rutherford et al. [84] and lines indicate the approximate positions of the different rainfall zones of South Africa: Solid line indicates the winter rainfall zone; dashed line indicates all year rainfall zone; and the rest of South Africa receives summer rainfall. The key to population acronyms are found in Table 1. Click here for file

Additional file 2: Table S2 Climatic variables for each population and the mean detection distances for prey and background vertical targets (leafy vegetation edge). Detection ranges calculated from the method of Stilz and Schnitzler [110] (http://134.2.91.93/~peter/calculator/range.php). The size range of prey taken by *R. capensis* at De Hoop is 2-19 mm [67] and this covers the range of small, medium and large prey in [110]. Climatic data were obtained from the nearest weather stations and provided by the South African Weather Service. Click here for file

Additional file 3: Figure S2 The distribution of 39 unique haplotypes across the 11 populations of *Rhinolophus capensis* sampled in this study. Key to acronyms given in Table 1. Click here for file

Additional file 4: Table S2 Genetic variability in 11 populations of *Rhinolophus capensis* based on 519 bp of the mitochondrial control region. Haplotype diversity (*Hd*), number of haplotypes, nucleotide diversity (*π*) and number of polymorphic sites are shown. Click here for file

Additional file 5: Figure S3 Neighbour-net network based on *p*-corrected distances of the 39 unique haplotypes isolated in this study. Each circle represents a unique haplotype coloured according to the biome/s in which it occurs. Pie graphs indicate where haplotypes are shared across several biomes. Click here for file

Additional file 6: Table S3 Lower and upper profile likelihood percentiles of *M*, the number of immigrants per generation scaled by mutation rate, calculated in Migrate-N (Beerli [120]). Click here for file
