# Supplementary figures and images for: Sensory trait variation in an echolocating bat suggests roles for both selection and plasticity
Source: BMC Evol Biol. 2014 Mar 27;14:60. doi: 10.1186/1471-2148-14-60 (PMC3986686; doi:10.1186/1471-2148-14-60)

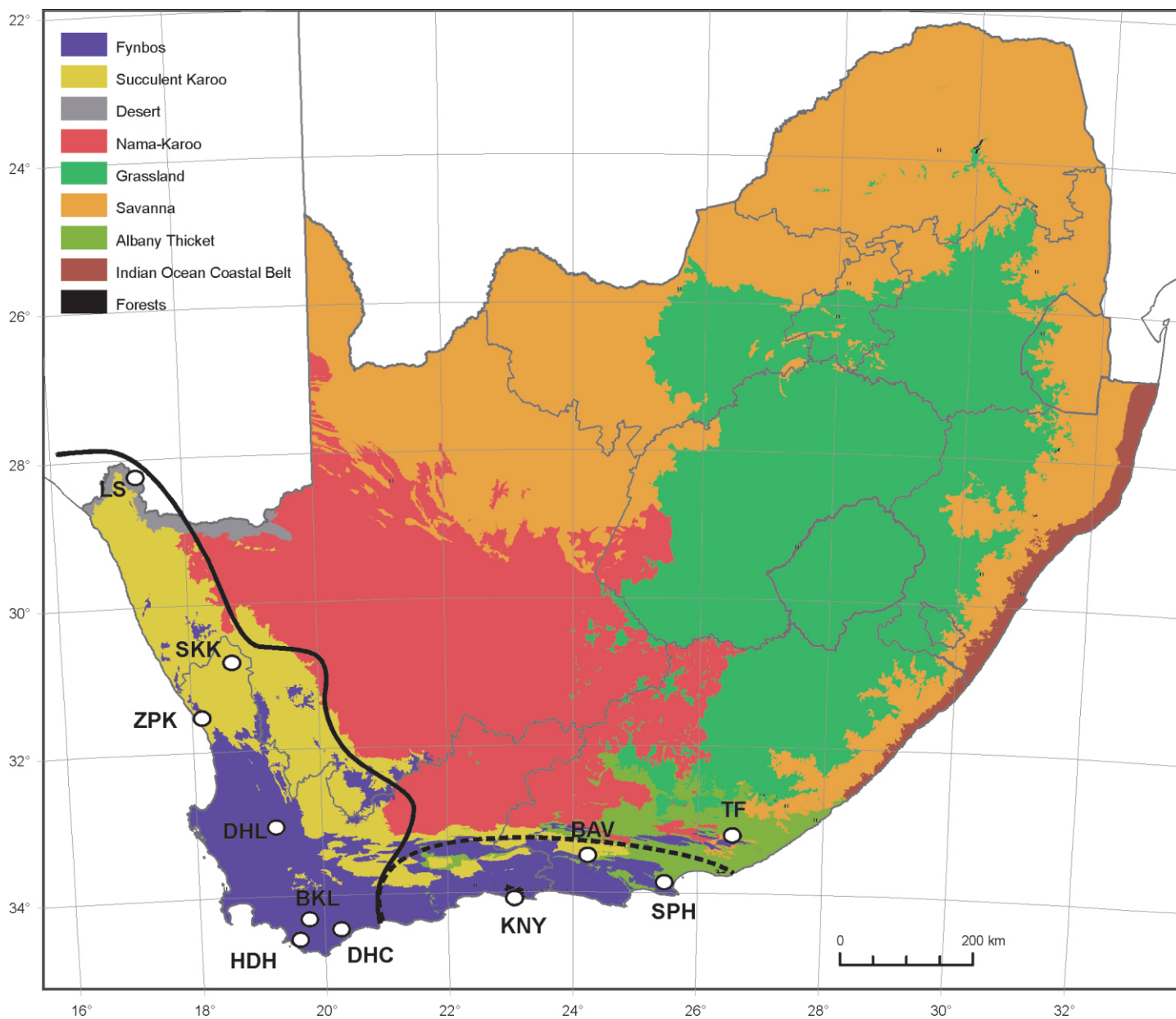

Additional file 1

Supplement: Additional file 1: Figure S1 — Map of the biomes of South Africa together with the geographic locations of the 11 populations of Rhinolophus capensis sampled in this study Biomes are from Rutherford et al. [84] and lines indicate the approximate positions of the different rainfall zones of South Africa: Solid line indicates the winter rainfall zone; dashed line indicates all year rainfall zone; and the rest of South Africa receives summer rainfall. The key to population acronyms are found in Table 1. [file 1471-2148-14-60-S1.pdf]

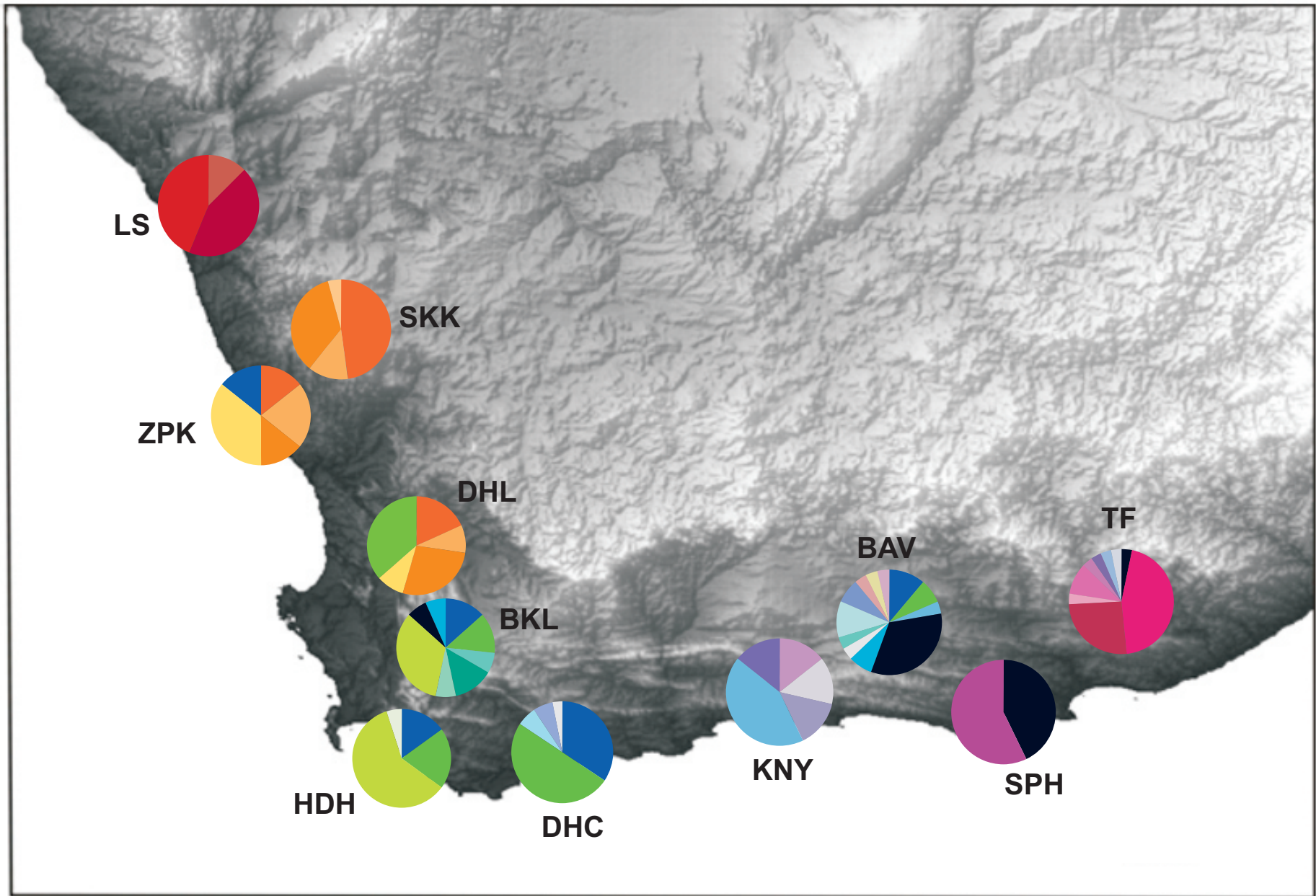

Additional file 3

Supplement: Additional file 3: Figure S2 — The distribution of 39 unique haplotypes across the 11 populations of Rhinolophus capensis sampled in this study. Key to acronyms given in Table 1. [file 1471-2148-14-60-S3.pdf]

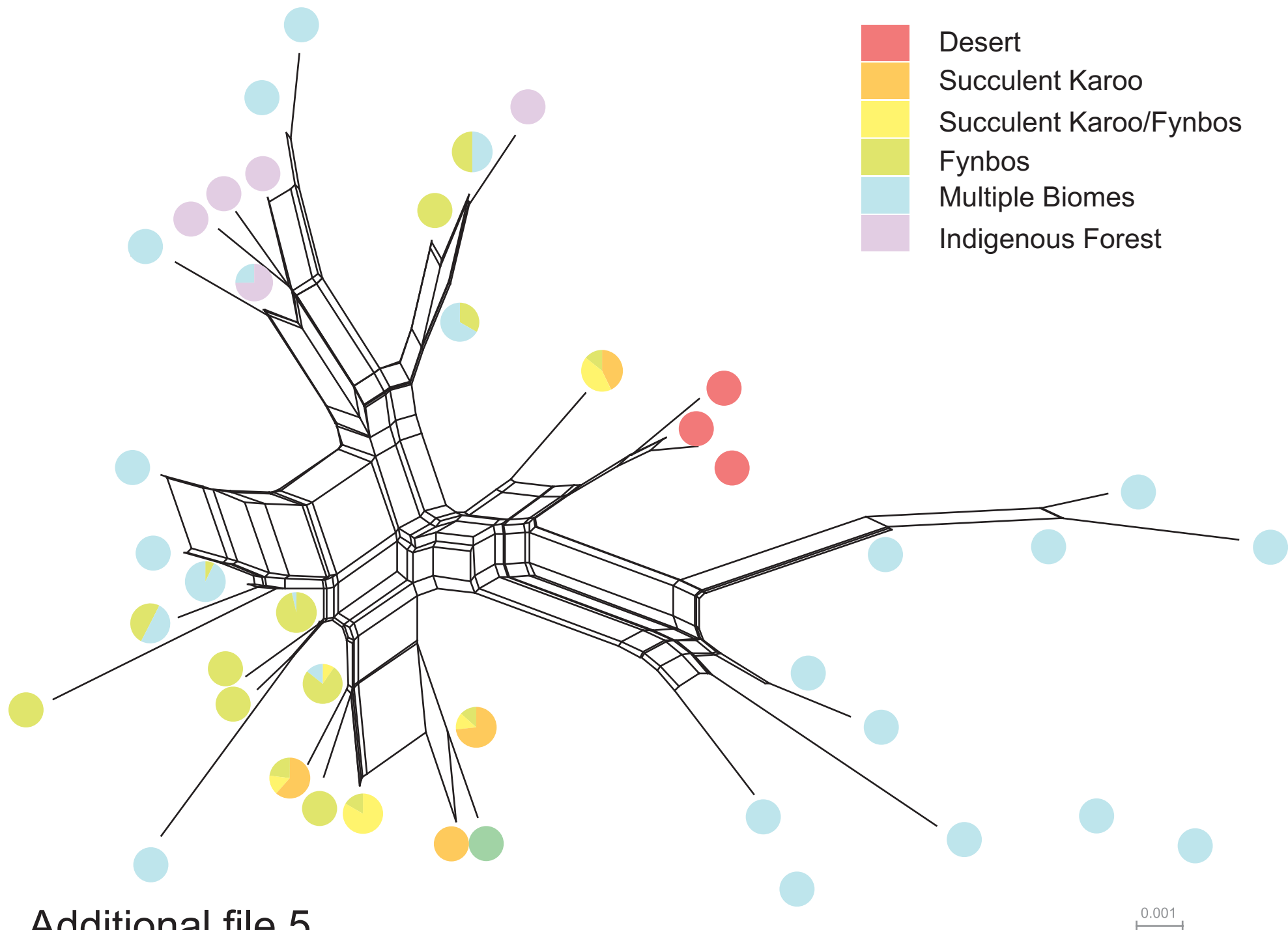

Additional file 5

Supplement: Additional file 5: Figure S3 — Neighbour-net network based on p-corrected distances of the 39 unique haplotypes isolated in this study. Each circle represents a unique haplotype coloured according to the biome/s in which it occurs. Pie graphs indicate where haplotypes are shared across several biomes. [file 1471-2148-14-60-S5.pdf]
