# Supplementary material for: Sensory trait variation in an echolocating bat suggests roles for both selection and plasticity
Source: BMC Evol Biol. 2014 Mar 27;14:60. doi: 10.1186/1471-2148-14-60 (PMC3986686; doi:10.1186/1471-2148-14-60)
Supplement: Additional file 2: Table S2 — Climatic variables for each population and the mean detection distances for prey and background vertical targets (leafy vegetation edge). Detection ranges calculated from the method of Stilz and Schnitzler [110] (http://134.2.91.93/~peter/calculator/range.php). The size range of prey taken by R. capensis at De Hoop is 2-19 mm [67] and this covers the range of small, medium and large prey in [110]. Climatic data were obtained from the nearest weather stations and provided by the South African Weather Service. [file 1471-2148-14-60-S2.docx]

**Additional file 2: Table S2 –Climatic variables for each population and the mean detection distances for prey and background vertical targets (leafy vegetation edge).**

Detection ranges calculated form the method of Stilz and Schnitzler [110] (http://www.biosonarlab.unituebingen.de/rangecalculator/index.html). The size range of prey taken by *R. capensis* at De Hoop is 2-19mm [67] and this covers the range of small, medium and large prey in [110]. Climatic data were obtained from the nearest weather stations and provided by the South African Weather Service.

| Population | Min temperature | Relative humidity | Detection distance (m) | | | |
| --- | --- | --- | --- | --- | --- | --- |
|  | (°) | (%) | small | medium | large | vegetation edge |
| LS | 12.05 | 73.31 | 1.5 | 2.7 | 3.7 | 8.1 |
| SKK | 11.24 | 82.27 | 1.5 | 2.6 | 3.6 | 7.6 |
| ZPK | 11.24 | 82.27 | 1.5 | 2.6 | 3.6 | 7.5 |
| DHL | 11.36 | 78.30 | 1.5 | 2.6 | 3.6 | 7.6 |
| BKL | 10.81 | 85.33 | 1.4 | 2.6 | 3.5 | 7.3 |
| HDH | 13.66 | 81.95 | 1.4 | 2.4 | 3.3 | 6.6 |
| DHC | 13.49 | 83.90 | 1.4 | 2.4 | 3.3 | 6.6 |
| KNY | 13.31 | 83.14 | 1.4 | 2.4 | 3.3 | 6.6 |
| BAV | 12.18 | 81.19 | 1.4 | 2.5 | 3.4 | 6.9 |
| SPH | 13.11 | 79.74 | 1.4 | 2.5 | 3.4 | 6.8 |
| TF | 11.20 | 78.17 | 1.4 | 2.6 | 3.5 | 7.3 |
