# Supplementary material for: Sensory trait variation in an echolocating bat suggests roles for both selection and plasticity
Source: BMC Evol Biol. 2014 Mar 27;14:60. doi: 10.1186/1471-2148-14-60 (PMC3986686; doi:10.1186/1471-2148-14-60)
Supplement: Additional file 4: Table S2 — Genetic variability in 11 populations of Rhinolophus capensis based on 519 bp of the mitochondrial control region. Haplotype diversity (Hd), number of haplotypes, nucleotide diversity (π) and number of polymorphic sites are shown. [file 1471-2148-14-60-S4.docx]

**Additional file 4: Table S2 – Genetic variability in 11 populations of *Rhinolophus capensis* based on 519bp of the mitochondrial control region.**

Haplotype diversity (*Hd*), number of haplotypes, nucleotide diversity (*π*) and number of polymorphic sites are shown

| Population | Sample size (n) | *Hd* | Number of haplotypes | π | Polymorphic sites |
| --- | --- | --- | --- | --- | --- |
| LS | 16 | 0.64 | 3 | 0.001 | 2 |
| SKK | 23 | 0.66 | 4 | 0.004 | 7 |
| ZPK | 14 | 0.82 | 5 | 0.005 | 7 |
| DHL | 11 | 0.82 | 5 | 0.005 | 7 |
| BKL | 15 | 0.88 | 8 | 0.007 | 10 |
| HDH | 20 | 0.61 | 4 | 0.006 | 8 |
| DHC | 32 | 0.64 | 5 | 0.003 | 11 |
| KNY | 7 | 0.86 | 5 | 0.004 | 8 |
| BAV | 27 | 0.87 | 12 | 0.007 | 18 |
| SPH | 7 | 0.57 | 2 | 0.006 | 6 |
| TF | 31 | 0.74 | 9 | 0.010 | 22 |
| All populations | 203 | 0.94 | 39 | 0.009 | 34 |
