# Supplementary material for: Sensory trait variation in an echolocating bat suggests roles for both selection and plasticity
Source: BMC Evol Biol. 2014 Mar 27;14:60. doi: 10.1186/1471-2148-14-60 (PMC3986686; doi:10.1186/1471-2148-14-60)
Supplement: Additional file 6: Table S3 — Lower and upper profile likelihood percentiles of M, the number of immigrants per generation scaled by mutation rate, calculated in Migrate-N (Beerli [120]). [file 1471-2148-14-60-S6.docx]

**Additional file 6: Table S3 – Lower and upper profile likelihood percentiles of *M*, the number of immigrants per generation scaled by mutation rate, calculated in Migrate-N (Beerli 2009).**

| Source population | Receiving population | Lower percentile (0.05) | Upper percentile (0.95) |
| --- | --- | --- | --- |
| SKK | LS | 8.78E-08 | 134.25 |
| ZPK | SKK | 1725.14 | 4921.60 |
| DHL | ZPK | 70.95 | 379.21 |
| ZPK | DHL | 1840.58 | 5953.23 |
| BKL | DHL | 1531.97 | 5384.67 |
| BAV | BKL | 97.47 | 434.03 |
| BKL | HDH | 1075.43 | 3250.20 |
| BKL | DHC | 104.18 | 464 |
| BAV | KNY | 44.12 | 493.25 |
| DHC | BAV | 917.74 | 2097.03 |
| KNY | BAV | 86.68 | 607.70 |
| BAV | SPH | 68.31 | 763.70 |
| BAV | TF | 115.85 | 515.85 |
